# Incidence and Predictors of New-Onset Silent Atrial Fibrillation after Coronary Artery Bypass Graft Surgery

**DOI:** 10.1155/2015/703685

**Published:** 2015-07-28

**Authors:** Charles Guenancia, Charline Pujos, Frederique Debomy, Ghislain Malapert, Gabriel Laurent, Olivier Bouchot

**Affiliations:** ^1^University Hospital, Department of Cardiology, 21000 Dijon, France; ^2^INSERM, U866, LPPCM, 21000 Dijon, France; ^3^University Hospital, Department of Cardiothoracic Surgery, 21000 Dijon, France; ^4^CNRS, UMR 5158, Le2I, 21000 Dijon, France

## Abstract

*Aims*. We investigated the incidence, risk factors, and prognostic impact of silent atrial fibrillation (AF) after coronary artery bypass graft (CABG) surgery. *Methods*. This observational study prospectively included 100 patients referred for CABG surgery. Holter ECG monitoring was used to record every arrhythmic event for 7 days. AF was defined as at least one episode >30 s. Episodes recorded on Holter ECG monitoring but not clinically identified were classified as silent AF. *Results*. Among 34 patients who developed new-onset AF, 13 had silent AF. Compared with patients with maintained sinus rhythm (SR), silent AF patients had a significantly higher logistic EuroSCORE (2.9 (1.5–5.2) versus 2.3 (1.4–3.7), *p* = 0.017) and were more likely to have previous sleep apnea (31% versus 8%, *p* = 0.016) and left atrial diameter >45 mm (36% versus 5%, *p* = 0.002). At one-year follow-up, 30% of silent AF patients had developed symptomatic AF versus 7% in the SR group (*p* = 0.03) and 11% in the clinical AF group (*p* = 0.21). *Conclusion*. After CABG surgery, silent AF is common and may be associated with a higher incidence of recurrences at one-year follow-up than clinical AF. Improved screening for silent AF may help to reduce thromboembolic events in this high-risk population.

## 1. Introduction

Atrial fibrillation is the most common cardiac arrhythmia, and its prevalence is growing with the ageing of the population [1, 2]. Postoperative atrial fibrillation (POAF) has a high prevalence, affecting 20 to 45% of coronary artery bypass graft surgery (CABG) patients within 7 days of the procedure [3, 4]. POAF is associated with increased duration of hospitalization, with high healthcare costs, and has been associated with an increased incidence of stroke, the need for a permanent pacemaker, and early and late mortality [5, 6].

Silent AF is defined as nonsymptomatic AF, not diagnosed by clinicians during hospitalization [7]. Although silent AF is mostly underdiagnosed, previous authors have shown that it was much more frequent than symptomatic AF after acute myocardial infarction [2]. Silent AF may adversely affect quality of life and is associated with a higher risk of cardiac events, such as acute heart failure [8], and with thromboembolic events (mainly stroke) [9]. However, in the particular setting of cardiac surgery, the incidence and the associated factors of silent AF have not yet been studied. Considering previous findings in acute myocardial infarction [2], stroke [10], and septic shock (citations), we hypothesized that long-duration Holter ECG monitoring after cardiac bypass surgery could unmask underdiagnosed silent AF.

This observational study was designed to characterize the incidence of post CABG silent AF, to determine its associated factors, and to quantify its impact on AF recurrence at one year.

## 2. Material and Methods

### 2.1. Study Design

The independent ethics committee of Dijon University Hospital approved the study protocol (CPP Est 1 2010–15; 29/03/2010) and written informed consent was obtained from the patients. The study was designed as a prospective observational study. All investigations were conducted in accordance with the principles outlined in the Declaration of Helsinki.

### 2.2. Selection of Patients

All of the patients undergoing CABG surgery at Dijon University Hospital from September 2011 to March 2013 were screened for participation in this prospective, observational study. The exclusion criteria were age <18 years or >80 years, previous atrial fibrillation/flutter, previous treatment with amiodarone, and previous cardiac surgery and emergency surgery. This observational study was designed to recruit the first 50 patients planned for on-pump (ONP) surgery and the first 50 patients planned for off-pump (OFP) surgery among patients meeting the inclusion criteria. This recruiting strategy has already been used in the particular setting of CABG [11]. The surgical strategy (ONP versus OFP) was decided by the medical and surgical team according to the anatomy of the coronary arteries.

### 2.3. Data Collection

Exhaustive clinical data were collected at admission and the following variables were recorded: age, sex, cardiovascular risk factors, cardiovascular and pulmonary diseases, previous regular medication, and preoperative echocardiographic parameters. Medications administered during the hospital stay (but not the doses) were recorded. Previous renal failure was defined as preoperative estimated glomerular filtration rate (eGFR) <60 mL/min using the MDRD formula. After discharge, each patient's daily medical files and treatment sheets were analyzed by a physician unaware of the Holter ECG monitoring results.

### 2.4. Transthoracic Echocardiography

All the patients underwent preoperative transthoracic echocardiography. The left ventricular ejection fraction (LVEF) was calculated using the Simpson method and was dichotomized at 45%. Left atrial (LA) anteroposterior diameter was dichotomized at 45 mm following classical cut-off values [12].

### 2.5. Anesthesia and Heart Surgery Procedure

Routine cardiac medications were continued until the morning of the surgery, except for clopidogrel, which was stopped at least 5 days earlier. Before the induction of anesthesia, a complete hemodynamic monitoring system was set up in the operating room. Off-pump surgery was performed with the use of Cor vasc (Coroneo, Montréal, Canada) to stabilize the heart. Patients were heparinized with 150 IU/kg intravenously to achieve an activated clotting time >300 s. The anastomoses were performed using temporary occlusion with LeGoo gel [13] (Sanofi, Paris, France).

On-pump surgery was performed in normothermia, with the use of aortic cross-clamping and anterograde warm blood cardioplegia. Patients were heparinized with 300 IU/kg to achieve an activated clotting time >400 s. Heparin was neutralized with 1 mg protamine sulfate per 100 IU given. During the study, there were no changes in the 2 surgical techniques.

### 2.6. Diagnosis of New-Onset AF

All patients underwent Holter ECG monitoring (Spider Flash, Sorin Group France) started immediately after the surgery. The Holter ECG device was programmed to record every arrhythmic event for 7 days and was removed after 7 days of recording or at death (whichever occurred first). AF was diagnosed according to the European Society of Cardiology guidelines; that is, any arrhythmia that presents the ECG characteristics of AF and lasting at least 30 s on a rhythm strip should be considered AF [14]. Silent AF was defined as the occurrence of AF on the Holter ECG recording in the absence of any mention of AF in the medical file during the first 7 days of the hospital stay. Holter ECGs were analyzed after hospital discharge. In contrast, clinical AF (whether symptomatic or not) was defined as any AF episode diagnosed by a physician during the hospital stay. The patients were usually monitored in the ICU for 2 to 3 days after cardiac surgery and then transferred to the ward, where an ECG was done once daily and heart rate and blood pressure were measured every 4 hours. In cases of any disturbance of the heart rate, an ECG was done.

Overall AF duration episodes were collected, and the total time in AF per day (AF burden) was calculated.

### 2.7. Treatment of New-Onset AF

In cases of clinical new-onset AF, patients received amiodarone and anticoagulants according to the CHAD_2_SVAS_2_C score [15]. As silent AF is not yet recognized as an entity that requires treatment accordingly to current AF therapy guidelines, and as the study was designed to be an observational one (i.e., with no intervention), the management of patients was not influenced by the Holter monitoring results.

### 2.8. Clinical Follow-Up

We recorded clinical outcomes over one-year of follow-up, namely, length of stay in the ICU and in the hospital and death in the ICU, at discharge (or at day 28, whichever came first), and at one year. The presence of new-onset AF and the occurrence of stroke between discharge and the follow-up time points were recorded. Follow-up information at one year was obtained by phone call to the patient, the patient's family, or the general practitioner. One-year follow-up was obtained for all patients.

### 2.9. Statistical Analysis

Continuous variables are presented as means ± standard deviations (SD) when normally distributed or medians and ranges otherwise, and categorical variables as numbers (percentages). Patients were classified into the clinical AF, silent AF, or sinus rhythm (SR) group according to the onset of atrial fibrillation on the Holter tracings and to the medical records. An exploratory identification of the characteristics of both groups was performed using the one-way ANOVA for continuous variables with Tukey post hoc analysis as appropriate and Fisher's exact test for categorical variables. All the tests were two-sided, and a *p* value <0.05 was considered significant. All analyses were performed using SPSS 20.0 0 (SPSS, Inc., Chicago, IL, USA).

## 3. Results

### 3.1. Patients' Characteristics

**Figure 1:**
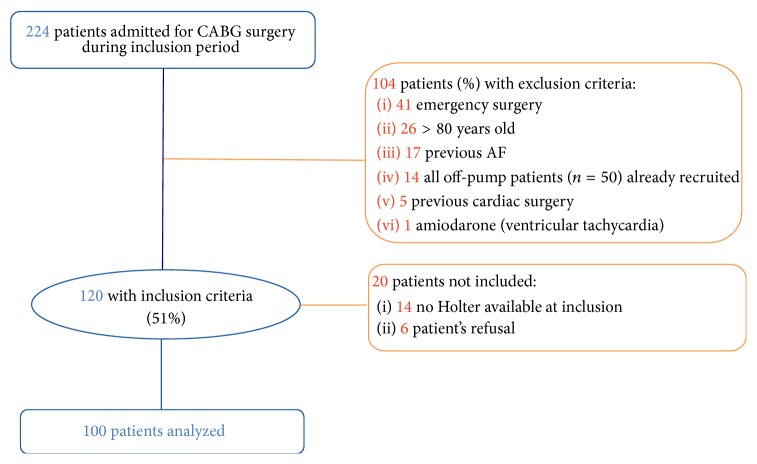
Flow chart of patient selection. AF: atrial fibrillation; CABG: coronary artery bypass graft surgery.

One hundred patients were prospectively included in the study (Figure 1). Among, them 21 (21%) developed clinical AF and 13 (13%) developed silent AF as detected by Holter monitoring.  Table 1 summarizes the baseline characteristics of the population. Patients who underwent the ONP surgery were comparable to OFP patients for all baseline characteristics except for a lower number of coronary artery grafts per procedure in the OFP group (OFP: 2.8 ± 0.8 versus ONP: 3.4 ± 0.9, *p* < 0.001).

When compared with maintained sinus rhythm patients (SR), clinical AF patients were significantly older (67 ± 9 versus 63 ± 9 years, *p* < 0.05), were more likely to have an impaired LVEF <45% (6 (29%) versus 2 (3%), *p* < 0.05), and were less often smokers (2 (10%) versus 25 (38%), *p* < 0.05).

When compared with SR patients, silent AF patients had a significantly higher logistic EuroSCORE (2.9 (1.5–5.2) versus 2.3 (1.4–3.7), *p* < 0.05), were more likely to have been diagnosed with sleep apnea syndrome (4 (31%) versus 5 (8%), *p* < 0.05), and were more likely to have LA enlargement (diameter > 45 mm) (4 (36%) versus 3 (5%), *p* < 0.05).

However, there was no significant difference between the clinical AF group and the silent AF group regarding baseline characteristics. Moreover, the preoperative CHAD2DS2-VASc score did not differ significantly between the three groups.

### 3.2. Surgery and ICU Management

Table 2 summarizes preoperative and postoperative characteristics. Although the number of grafted coronary arteries was similar between the three groups, aortic cross-clamp and total cardiopulmonary bypass (CPB) duration was longer in silent AF patients than in SR or clinical AF patients. When compared with SR patients, silent AF patients had a longer CPB duration (119 (103–145) versus 89 (75–109) minutes, *p* < 0.05) and a longer aortic cross-clamp duration (96 ± 21 versus 71 ± 22 minutes, *p* < 0.05). CPB and aortic cross-clamp durations were longer in silent AF patients than in clinical AF patients (119 (103–145) versus 95 (75–113) minutes, *p* < 0.05; 96 ± 21 versus 71 ± 19 minutes, *p* < 0.05, resp.).

Concerning postoperative characteristics, inotropic catecholamines, amiodarone, and vitamin K oral anticoagulants (VKA) were more often given to clinical AF patients than to SR patients (6 (29%) versus 5 (8%), *p* < 0.05, 14 (67%) versus 13 (20%), *p* < 0.05, and 8 (40%) versus 0, *p* < 0.05, resp.). Furthermore, clinical AF patients were more likely than SR patients to suffer from infectious complications (3 (14%) versus 1 (2%), *p* < 0.05).

### 3.3. Holter Recordings Analysis

Table 3 summarizes data from 7-day-Holter monitoring, according to the occurrence of clinical or silent AF. Patients with clinical AF had a higher mean heart rate than patients with SR (79 ± 10 versus 74 ± 9 bpm, *p* < 0.05). Both AF groups had a similar mean heart rate (79 ± 10 versus 74 ± 8 bpm, *p* < 0.05). The onset of the first AF episode was significantly later in the silent AF group than in the clinical AF group, within the 4 first days of monitoring (7 (54%) versus 21 (100%), *p* = 0.001). Not surprisingly, the AF burden during the first seven days of recording was shorter in the silent than in the clinical AF group (35 (3–120) versus 869 min (161–2366), *p* = 0.001).

### 3.4. Follow-Up

At one-year follow-up, symptomatic (paroxysmal or persistent) AF was more frequently diagnosed in patients from the silent AF group than those from the SR group (30% versus 7%,  *p* = 0.023). These patients were also more often on oral anticoagulants (20% versus 3%, *p* = 0.03). Two ischemic strokes occurred within the first year of follow-up, one in the SR group and one in the silent AF group. However, the small number of events did not allow a statistical comparison between the three groups regarding stroke or death at one year.

## 4. Discussion

Our observational study was designed to characterize underdiagnosed silent AF in a post-CABG setting. We also aimed to identify risk factors for silent AF and its prognostic impact at one-year follow-up. The main results of our work are as follows:

- Silent AF is common after cardiac surgery (13% of all patients, 34% of AF patients).
- The risk profile in patients who develop silent AF after CABG is different from that in SR or clinical AF patients, suggesting that silent AF may be triggered by other mechanisms.
- Despite the small number of events, silent AF patients were more likely to develop symptomatic AF at one year than were SR or clinical AF patients. These results highlight the importance of screening for silent AF, which may eventually become a clinically relevant arrhythmia.

Silent AF was defined as nonsymptomatic AF and AF not diagnosed by clinicians during the hospitalization. Its incidence is underestimated as it can only be identified by several days of continuous cardiac monitoring using a Holter ECG or with implantable loop recorders. In fact, silent AF is mostly underdiagnosed because of its short duration and its lack of associated symptoms. Patients with silent AF may therefore not receive appropriate anticoagulation therapy, and the disease may be unmasked by a cerebrovascular event as its first manifestation [8]. Previous studies have shown that approximately 25% to 30% of patients with stroke had AF that was not previously recognized [16]. Recently, Stamboul et al. demonstrated that silent AF is three times more frequent than symptomatic AF after acute myocardial infarction [2]. In the setting of septic shock, one-third of new-onset AF was defined as silent AF [17]. However, even though silent AF is not symptomatic, the onset of this arrhythmia is associated with higher rates of cardiac adverse events like congestive heart failure and stroke [9].

When the Holter recordings of silent AF and clinical AF patients were compared, several significant differences were noted: the burden of AF in the silent AF group was lower than that in the clinical AF group during the Holter ECG monitoring, and in the silent AF group the onset of AF was more likely to occur after the first 4 days following the cardiac surgery. These results are closely linked to the definition of silent AF. It is obvious that the longer the AF episode lasts, the greater the chance it will be identified by heart rate monitoring, systematic ECG, or a clinical examination. Moreover, most patients were continuously monitored in the ICU during the first three days. We may then hypothesize that screening for AF was more intensive during the ICU stay than in the surgery ward. However, this hypothesis cannot explain the whole pattern of silent AF. Indeed, 6/13 of silent AF patients (46%) had the first onset of AF during their ICU stay. In fact, unlike Holter ECG recordings, tracings from ECG monitors are not usually reanalyzed afterwards. Besides, monitor alarms may not have been set up appropriately for the detection of AF as opposed to life-threatening arrhythmias like ventricular fibrillation (VF).

It has recently been demonstrated that early (within the first 5 days after cardiac surgery) and late (6 to 30 days after cardiac surgery) POAF have different predictors [18]. Notably, late POAF was associated with conventional risk factors of AF in the general population, whereas early AF was associated with low BMI, high CRP levels, and previous myocardial infarction. In our study, silent AF patients had significantly higher logistic EuroSCOREs than SR patients. This finding is probably linked to higher age, which is one of the major risk factors of AF [4]. Moreover, our study is in accordance with previous works regarding the association between the EuroSCORE and the onset of AF after cardiac surgery [19, 20]. We found that patients who developed silent AF were more likely than SR patients to have LA dilation (diameter > 45 mm). This finding is consistent with other studies suggesting that LA size could be a predictor of AF [21, 22]. Interestingly, previous obstructive sleep apnea has been associated with significant atrial remodeling characterized by atrial enlargement [23]. In our study, silent AF patients were more likely than SR patients to have previous obstructive sleep apnea. van Oosten et al. suggested that patients with previous obstructive sleep apnea may have intermittent hypoxia leading to atrial remodeling, and this phenomenon has been associated with the onset of AF [24]. Several other studies have also demonstrated that previous obstructive sleep apnea is a predictor of AF [25]. Silent AF was also associated with longer aortic cross-clamp duration compared with the SR and clinical AF groups. In keeping with our results, Bidar et al. recently found that aortic cross-clamp time was a strong predictor of 30-day POAF [18]. Interestingly, the authors carefully recorded any AF episode within the 30 days after cardiac surgery using a dedicated device for monitoring, and thus their results can be compared to ours in terms of silent AF identification rates. Moreover, the aortic cross-clamp time was no longer a predictor of POAF when they focused on early POAF predictors. This result suggests that late POAF (the definition of which was quite similar to that of silent AF, i.e., POAF occurring after day 6, recorded on an external 1-lead transtelephonic loop recorder with an autotrigger for AF) was associated with longer aortic cross-clamp times.

Finally, silent AF patients were more likely than SR patients and clinical AF patients to develop symptomatic AF at one year. We may hypothesize that clinical AF patients were more often on antiarrhythmic drugs, which may in turn have protected patients from AF recurrences. However, silent AF could also be interpreted as incidental AF diagnosed during the hospital stay thanks to the Holter monitoring, whereas clinical AF would more likely be related to the acute cardiac injury triggered by the surgery. Bidar et al. demonstrated that 30-day monitoring using a transtelephonic loop recorder, with an autotrigger algorithm for AF, improved its screening: 25% of total POAF recorded occurred between day 6 and day 30 [18]. This observation suggests that POAF occurs not only during the first postoperative days but also in the weeks and months thereafter. If this is the case, early discontinuation of oral anticoagulation may have adverse effects. Indeed, continuation of warfarin after discharge showed protective effects against long-term mortality [26]. These results highlight the importance of screening for silent AF and raise the issue of anticoagulation therapy to prevent thromboembolic events. A randomized study comparing systematic anticoagulation therapy in silent and clinical AF during the hospital stay with the classical strategy is required to assess the potential benefit of improved AF screening.

### 4.1. Study Limitations

Our study has several limitations. First of all, given the relatively small number of patients (since our inclusion criteria were designed to recruit a homogeneous and “otherwise healthy” cohort of patients), our results should be confirmed in larger population studies. Another limitation must be highlighted: even though the patients undergoing on-pump and off-pump surgery were comparable, the study was not designed as a randomized controlled trial to compare the incidence of POAF in these two groups. Moreover, the assessment of LA dimensions could have been improved by the measurement of LA surface area and volume. As the follow-up data were recorded at one year, we did not collect the date of each event and we were unable to conduct survival analyses on our data.

## 5. Conclusion

Silent AF after CABG surgery is common (1/3 of recorded AF) and mostly underdiagnosed even with the use of ECG monitors in the ICU. We have shown in this study that silent AF has to be considered for appropriate medications (especially anticoagulant therapy according to the CHAD2SVASC2 score), as it is associated with a higher incidence of recurrences at one year than is the case for clinical AF. Although this is a small observational study, we think that it is relevant to improving AF screening during hospital stays by using Holter monitoring in order to prevent thromboembolic events in this high-risk population.

## Figures and Tables

**Table 1:** Baseline characteristics according to the occurrence of atrial fibrillation.

*n* (%), median (interquartile range),	SR group	Silent AF group	Clinical AF group	*p*
mean ± SD	(*n* = 66)	(*n* = 13)	(*n* = 21)	
Risk factors				
Age, years	63 ± 9	65 ± 8	67 ± 9^**∗**^	0.09
Female sex	4 (8)	0 (0)	3 (14)	0.32
Systemic hypertension	43 (65)	8 (62)	13 (62)	0.95
Diabetes mellitus	25 (38)	5 (39)	6 (29)	0.73
Smoking	25 (38)	4 (31)	2 (10)^**∗**^	**0.05**
Obesity (BMI ≥ 30 kg/m²)	32 (48)	9 (70)	10 (48)	0.37
Hypercholesterolemia	50 (76)	10 (77)	17 (81)	0.89
Family history of CAD	28 (42)	7 (54)	11 (53)	0.61
Medical history				
Recent MI	18 (27)	7 (54)	8 (38)	0.15
Previous peripheral arteriopathy	18 (27)	5 (39)	4 (19)	0.46
Previous sleep apnea	5 (8)	4 (31)^**∗**^	4 (19)	**0.049**
Previous treatments				
Beta blockers	53 (80)	11 (85)	20 (95)	0.27
Calcium Channel Blockers	19 (29)	3 (23)	9 (43)	0.38
Diuretics	16 (24)	6 (46)	8 (38)	0.19
Statins	64 (97)	12 (92)	21 (100)	0.42
ACE inhibitors	54 (82)	10 (77)	18 (88)	0.81
VKA	3 (5)	1 (8)	1 (5)	0.89
Clinical data				
NYHA > 1	31 (47)	6 (46)	9 (43)	0.95
CCS > 3	5 (8)	0	2 (10)	0.54
LVEF (%)	57 ± 11	55 ± 9	54 ± 14	0.44
LVEF < 45%	2 (3)	1 (8)	6 (29)^**∗**^	**0.002**
EuroSCORE	3 (1–5)	4 (2–6.5)	3 (2–6.5)	0.18
Logistic EuroSCORE	2.3 (1.4–3.7)	2.9 (1.5–5.2)^**∗**^	2.2 (1.4–6)	0.27
LA diameter	39.5 (35–43)	44 (37–46)	41 (36–44)	0.11
LA diameter > 45 mm	3 (5)	4 (36)^**∗**^	3 (16)	**0.009**
CHAD2DS2-VASc score > 1	44 (67)	10 (77)	16 (76)	0.59
Serum creatinine, *μ*mol/L	88 (74–102.5)	88 (80–101.5)	90 (76.5–101.5)	0.98
NT pro-BNP, pg/mL	146 (61–491)	156 (98–1852)	136 (75–533)	**0.019**

ACE indicates angiotensin conversion enzyme; AF: atrial fibrillation; BMI: body mass index; CAD: Coronary Artery Disease; CCS: Canadian cardiovascular society; LA: left atrial; LVEF: left ventricular ejection fraction; min: minutes; MI: myocardial infarction; NYHA: New York heart association; SD: standard deviation, SR: sinus rhythm; VKA: vitamin K antagonists.

^**∗**^ *p* < 0.05 compared to SR group.

**Table 2:** Clinical characteristics according to the occurrence of atrial fibrillation.

*n* (%), median (interquartile range),	SR group	Silent AF group	Clinical AF group	*p*
mean ± SD	(*n* = 66)	(*n* = 13)	(*n* = 21)	
Surgery data				
Number of CABG				0.55
1	1 (2)	0	1 (5)	
2	11 (17)	3 (23)	7 (33)	
3	28 (42)	6 (46)	6 (29)	
4	22 (33)	3 (23)	5 (25)	
5	4 (6)	1 (8)	1 (5)	
6	0	0	1 (5)	
CPB	35 (53)	5 (39)	10 (48)	0.61
CPB duration (min)	89 (75–109)	119 (103–145)^*∗*§^	95 (75–113)	0.062
Aortic cross-clamp duration (min)	71 ± 22	96 ± 21^*∗*§^	71 ± 19	0.062
ICU management				
Mechanical ventilation duration (hours)	5 (4–7)	5 (4–6)	4 (3–5)	0.22
ICU stay duration (hours)	60 (27–91)	27 (19–91)	46 (24–89)	0.32
Norepinephrine	31 (47)	4 (31)	8 (38)	0.49
Inotropic catecholamine	5 (8)	3 (23)	6 (29)^*∗*^	**0.032**
Amiodarone	13 (20)	3 (23)^§^	14 (67)^*∗*^	**<0.001**
Biological data				
pH 3 hours after surgery	7.39 ± 0.06	7.38 ± 0.07	7.37 ± 0.05	0.22
pH 24 hours after surgery	7.37 ± 0.04	7.37 ± 0.04	7.37 ± 0.04	0.90
Troponin 3 hours after surgery, ng/mL	3.5 (0.9–5.8)	1.7 (0.5–6.7)	2.9 (0.4–6.3)	0.62
Troponin 24 hours after surgery, ng/mL	2.3 (1.7–5)	2 (1.2–5.3)	1.8 (1.1–3.4)	0.34
CRP peak, mg/L	194 ± 86	214 ± 84	197 ± 110	0.78
NT pro-BNP peak, pg/mL	150 (65–531)	136 (64–675)	168 (71–1947)	0.49
Lowest hemoglobin level, g/dL	9 (8.3–10)	9.1 (8.1–11.7)	9.6 (8.7–9.9)	0.42
In-hospital follow-up				
Acute kidney injury (KDIGO stage 1)	20 (30)	4 (31)	9 (43)	0.56
Sepsis	1 (2)	0	3 (14)^*∗*^	**0.025**
Stroke	0	0	0	—
Myocardial infarction	3 (5)	0	0	0.45
Treatment at discharge				
Betablockers	60 (91)	13 (100)^§^	15 (75)	0.055
Calcium Channel Blockers	8 (12)	1 (8)	3 (14)	0.82
Statins	36 (96)	11 (85)	18 (90)	0.32
ACE inhibitors	52 (82)	11 (86)	12 (57)	0.17
VKA	0	1 (8)^*∗*§^	8 (40)^*∗*^	**<0.001**

AF indicates atrial fibrillation; CABG: coronary artery bypass graft; CPB: cardio pulmonary bypass; CRP: C Reactive Protein; GDF: Grow Differentiation Factor; ICU: intensive care unit; NT Pro-BNP: N Terminal Pro-Brain Natriuretic Peptide; SD: standard deviation; SR: sinus rhythm; VKA: vitamin K antagonists.

^*∗*^ *p* < 0.05 compared to SR group; ^§^ *p* < 0.05 compared to clinical AF group.

**Table 3:** Rhythm Characteristics According To The Occurrence of Atrial Fibrillation.

*n* (%), median (interquartile range),	SR group	Silent AF group	Clinical AF group	*p*
mean ± SD	(*n* = 66)	(*n* = 13)	(*n* = 21)	
Holter ECG monitoring				
Mean Heart Rate, bpm	74 ± 9	74 ± 8	79 ± 10^*∗*^	0.12
Mean HRV	52 (39–65)	51 (29–66)	56 (38–75)	0.50
Heart Rate D1, bpm	78 ± 10	77 ± 10	82 ± 11	0.31
HRV D1	74 (50–98)	73 (49–92)	86 (46–117)	0.70
Heart Rate D7, bpm	70 (64–78)	70 (65–83)	71 (62–82)	0.77
HRV D7	83 (66–114)	131 (79–178)	87 (56–147)	0.31
NSVT	9 (14)	3 (23)	4 (19)	0.78
AF				
Time between admission and first AF record (hours)	—	2 (1–5)	1 (1-2)	0.19
First AF onset within the 4 first days of monitoring	—	7 (54%)^§^	21 (100%)	0.001
Total duration of AF during Holter ECG monitoring (min)	—	35 (3–120)^§^	869 (161–2366)	0.001
Number of AF episodes during Holter ECG monitoring	—	1 (1-2)	2 (1–5)	0.29
Heart rate preceding first AF episode	—	77 (71–84)	81 (69–93)	0.62
Heart rate of first AF episode	—	129 (117–144)	131 (118–151)	0.41
Night AF	—	5 (39)	10 (48)	0.52

AF indicates atrial fibrillation; bpm: beats per minute; HRV: heart rate variability; NSVT: non-sustained ventricular tachycardia; SD: standard deviation; SR: sinus rhythm.

^*∗*^ *p* < 0.05 compared to SR group; ^§^ *p* < 0.05 compared to clinical AF group.
